# Secondary Outcomes of the Ole e 1 Proteins Involved in Pollen Tube Development: Impact on Allergies

**DOI:** 10.3389/fpls.2020.00974

**Published:** 2020-07-03

**Authors:** M. Fernández-González, E. González-Fernández, D. Fernández-González, F. Javier Rodríguez-Rajo

**Affiliations:** ^1^ CITACA, Agri-Food Research and Transfer Cluster, University of Vigo, Ourense, Spain; ^2^ Pole of the Faculty of Sciences, Earth Sciences Institute (ICT), University of Porto, Porto, Portugal; ^3^ Department of Biodiversity and Environmental Management (Botany), University of León, León, Spain; ^4^ Department of Natural, Environmental and Anthropic Hazards of Cultural Heritage, Institute of Atmospheric Sciences and Climate-CNR, Bologna, Italy

**Keywords:** Ole e 1, aeroallergens, pollen, ELISA, allergy risk days

## Abstract

Ole e 1 protein is involved in olive fertilization mechanisms controlling pollen tube development. Similarly to the process by which pollen grains hydrated and form a pollen tube upon arrival at the female gametophyte, when pollen grains fall on the nasal mucosa the expression of Ole e 1 protein induce allergic reaction in sensitive individuals. The research was conducted in Ourense (North-western Spain), during the 2009–2018 period. Ole e 1 protein was collected using a Cyclone Sampler and processed with the ELISA methodology. Airborne *Olea* pollen were monitored using a Hirst type volumetric sampler. Allergy risk episodes identified by pollen concentrations were detected in five of the 10 studied years, all with moderate risk. Actual risk episodes of allergy increased when the combination of pollen and Ole e 1 concentrations were considered. Moderate risk episodes were detected during 9 years and high-risk episodes during 3 years. In addition, some years of low annual pollen concentrations recorded high total amounts of Ole e 1. During the years with lower pollen production, the tree increases the synthesis of Ole e 1 to ensure proper pollen tube elongation in order to complete a successful fertilization. This fact could justify higher sensitization rates in years in which a lower pollen production is expected. The present method contributes to the determination of the real exposure to Ole e 1 allergen evaluating the role of this protein as an aeroallergen for sensitized population. The allergen content in the atmosphere should be considered to enhance the prevention of pollinosis clinical symptomatology and the reduction of medicine consumption.

## Introduction

The most important plant ecological event is reproduction, whose features follow different strategies to ensure a successful fertilization and the formation of new seeds to perpetuate the species. The pollen tube plays a fundamental role in sexual processes facilitating the displacement of the sperm cells through the style and ovary tissues to the ovule for fertilization (Mascarenhas, 1993). During this physiological phenomena, the expression of several proteins with different functions and signalling mechanisms is involved (Aloisi et al., 2017; Jimenez-Quesada et al., 2019). The isolation and characterization of those genes expressed during pollen tube formation is studied by different authors (Speranza et al., 2012; Del Duca et al., 2014; Lora et al., 2016; Jimenez-Quesada et al., 2019; Mandrone et al., 2019). The importance of the Ole e 1 protein is highlighted in the case of olive sexual fertilization (Alché et al., 1999). This protein is produced in gametophytic and sporophytic tissues during the ontogeny of the Oleaceae family pollen grains (Alché et al., 2004; Rodríguez-Rajo et al., 2010). Ole e 1 is localized in the tapetum cells and the orbicules during the trafﬁc of proteins from the tapetum cells to the microspores (Rodríguez-Rajo et al., 2010). At the mature stage of pollen grains, the protein is located in the pollen ectexine and the cytoplasm, as the synthesis of Ole e 1 occurs in the endoplasmic reticulum where the protein is also stored (Rodríguez-García et al., 1995; Alché et al., 2004).

During the moments before the start of the pollen tube emergence, significant increases of the protein are detected (Alché et al., 1999). Pollen grains conserve the metabolic mechanism at the dehydrated stage to reserve an important quantity of Ole e 1-like proteins, maybe waiting for the start of the pollen tube growth (Rodríguez-Rajo et al., 2010). At this moment, the allergen is detected in the internal subapical region of the pollen tube, especially in the endoplasmic reticulum cisternae, and in the pollen extracellular zone of pollen tube cell wall (Alché et al., 2004). A similar role is attributed to all Ole e 1-like proteins from different species (Barderas et al., 2005; Rodríguez-Rajo et al., 2010), as modifiers of the pollen grain cell wall at different ontogeny stages. Ole e 1-like proteins control pre-germination events, pollen tube emergence, signalling and guidance, and the maintaining of the osmotic gradient in the pollen tube (Alché et al., 2004). Moreover, the presence of the Ole e 1 in the pollen exine suggests that this protein would also be involved in pollen-stigma and pollen tube-style cells recognition processes (Alché et al., 2004).

A similar process than the occurred in the female gametophyte takes place when airborne pollen grains fall, under certain conditions, in the nasal mucosa of a sensitive patient (Fernández-González et al., 2010). The expression of the Ole e 1 allergen induces allergic reactions in humans. Due to the importance and extension of the olive cultivation along the Mediterranean basin, important rates of pollen sensitization up to 29.7% in allergic patients are recorded (Feo Brito et al., 2011). The northern limit of the olive tree distribution in the Iberian peninsula is the Eurosiberian bioclimatic region where only the 8% of the pollinosis people show positive effects to *Olea* pollen (Belmonte et al., 1998).

Twelve allergens are isolated and characterized in the olive pollen (Esteve et al., 2012). Common olive group 1 is the major allergen with 16-kDa, affecting more than 70% of sensitized people to olive pollen (Palomares et al., 2006). The main allergen has an amino acid sequence with a homology of more than 85% with the main allergens of other widely distributed family members such as lilac, privet, ash or forsythia (Batanero et al., 1994). *In vivo* and *in vitro* studies demonstrate the cross reaction between the members of the Oleaceae family through the use of Ole e 1 to detect Ole e 1-like proteins in the *Fraxinus* pollen microsporogenesis phases (Rodríguez-Rajo et al., 2010), and the Fra e 1 and Lig v 1 allergens in the atmosphere (Vara et al., 2016). In addition, Ole e 1 presents relevant homology rates, around 24–34%, with pollen allergens from maize, tomato, ryegrass, birch, rice and *Arabidopsis* (Lombardero et al., 1994).

The aim of this study is to evaluate whether a protein involved in the olive fertilisation mechanisms controlling pollen tube development, such as Ole e 1, could be detected in the atmosphere acting as an important aeroallergen for sensitized people. This information will allow us to enhance the prevention of pollinosis clinical symptomatology and the reduction of medicine consumption.

## Material and Methods

### Area and Period of Study

The research was conducted in the North-western Spain, in Ourense (42°0’N 7°5’W) during a 10 years period, from 2009 to 2018. The area of the study was placed at the limit of the distribution of the *Olea europea* L. tree, in the boundary between the bioclimatic Mediterranean and Eurosiberian regions, characterized by an annual mean temperature of 14°C and a quantity of 772 mm as total rainfall average (Martínez-Cortizas and Pérez-Alberti, 1999). Small number of old olive trees were present in the gardens of the city of Ourense, however, new extensions of olive trees of 300 ha were planted during the last ten years throughout southern Galicia around the area of study.

### Aeroallergen Sampling

The Ole e 1 content in the airborne aerosol was collected by means of a Multi-Vial Cyclone Sampler (Burkard Manufacturing Co Ltd.). The device was located in the roof of the Polytechnic building of Ourense campus. The Cyclone is a continuously wind-oriented sampler that generates a single reverse-flow cyclone to capture the aeroallergens under a 16 L/min airflow rate. Daily samples were captured in Eppendorf vials from the end of April to June. The analysis was conducted by applying the Takahashi et al. (2001) method modified by Moreno-Grau et al. (2006). The four steps 2-site ELISA methodology was used to quantify the Ole e 1 content in the bioaerosol (Fernández-González et al., 2010; Vara et al., 2016). Primary mouse anti-Ole e 1 monoclonal antibody 5A3 L-121, purified natural Ole e 1 antibody and a biotinylated rabbit anti-Ole e 1 polyclonal antibody were used (Roxall Medicina España S.A.). Absorbance measurements at 492 nm were conducted.

### Pollen Monitoring

To complete the study, *Olea* pollen was monitored in the atmosphere during the same period than Ole e 1 proteins. The sampling was performed using a Hirst type LANZONI VPPS 2000 volumetric sampler with a continuous suction flow rate of 10 L/min, which represent the average human breath per minute. The device was placed at 2 m from the allergen trap. An optical microscope with a magnification of 400× was used for the identification of the pollen grains (Galán et al., 2007). The main pollen season (MPS) was calculated considering the period including the 95% of pollen recorded during a year. MPS starts when the accumulated sum of pollen attained the 2.5% of the year and finished the day when the 97.5% of pollen was achieved.

### Allergy Risk Days Assessment

The number of days in which sensitized people could be more likely to develop pollen related allergic symptomatology caused both, for pollen and allergens, was assessed by means of a linear regression equation between pollen and allergen data. The olive risk thresholds described by the Spanish Aerobiology Network (Galán et al., 2007) were applied to establish the risk Ole e 1 threshold: Low (1–50 pollen/m^3^), Moderate (51–200 pollen/m^3^) and High (>200 pollen/m^3^). Pollen Allergen Potency (AP) index was calculated for each taxa, which represented the rate between the allergen and pollen grain concentrations. The STATISTICA 7 program was used for the statistical analysis.

## Results

**Figure 1:**
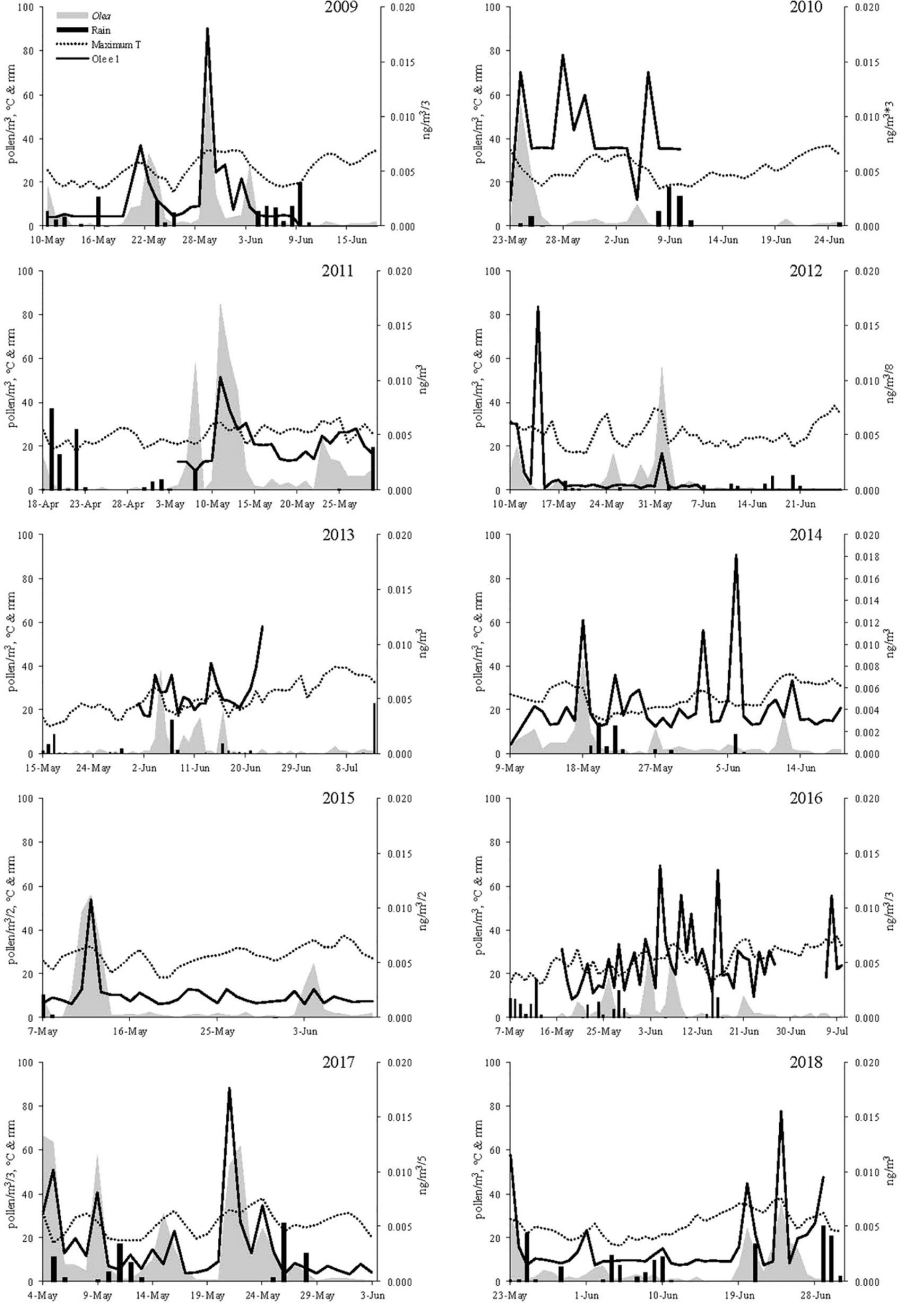
Pollen grains (grey area), Allergen concentration (black line), Maximum Temperature (black points) and Rainfall (black bars) during the study period.

Ole e 1 aeroallergen concentrations were detected over a period of 10 years in northwestern Spain. The evolution of aeroallergen and pollen concentrations, precipitations and maximum temperature were shown in **Figure 1**. Olive trees flowering occurred during spring months, mainly form early May to late June. In general allergen values matched airborne pollen concentrations, but some mismatches between pollen and aeroallergen presence in the air were detected. The features of the flowering period were shown in **Table 1**. The onset of the Main Pollen Season (MPS) was recorded as average on May12th and the final date on June 21th. The earliest start date was registered on April 18th 2011, and the latest on May 23th 2018. The flowering period was lengthy, recording a mean duration of 44 days throughout the data set, ranging from the 31 days in 2107 to the 65 registered in 2106. During recent years, a not significant trend to a delay of the MPS start date was detected. The annual pollen integral—API—(calculated as the sum of the daily average concentrations during the MPS) accounted for an average of 377 pollen/m^3^, ranging from the 112 detected in 2010 to the 1,388 recorded in 2017. The total annual Ole e 1 aerollergen amount accounted for an average of 0.247 ng/m^3^, ranging from the 0.047 ng detected in 2010 to the 0.711 ng recorded in 2016. In recent years, a trend to the increase of total pollen and allergen amount was registered. Pollen and allergen daily peaks during the study period were observed on May 4th 2017 with 199 pollen/m^3^ and on May 14th 2012 with 0.134 ng/m^3^, respectively. Pollen Allergen Potency (AP), considered as the ratio between pollen and allergen concentrations, was evaluated. An average of 0.0008705 ng/pollen was registered. The highest value was 0.0034351 ng/pollen during the year 2016, while the lowest was reached in the year 2011 with 0.0002362 ng/pollen (**Table 1**).

**Table 1:** Start, end dates and length of the *Olea* main pollen season (MPS) over the study period.

		2009	2010	2011	2012	2013	2014	2015	2016	2017	2018	Average
**MPS**	**Start**	10-May	23-May	18-Apr	10-May	15-May	9-May	7-May	7-May	4-May	23-May	**12-May**
	**End**	18-Jun	25-Jun	29-May	27-Jun	13-Jul	19-Jun	10-Jun	10-Jul	3-Jun	1-Jul	**21-Jun**
	**Length**	40	34	42	49	60	42	35	65	31	40	**44**
**Pollen**	**Total**	239	112	426	395	146	179	447	207	1388	232	**377**
	**Peak day**	65	55	85	112	38	42	112	33	199	35	**78**
	**Date peak**	29-May	24-May	11-May	1-Jun	5-Jun	18-May	12-May	3-Jun	4-May	24-Jun	**25-May**
**Ole e 1**	**Total**	0.223	0.047	0.101	0.337	0.127	0.174	0.141	0.711	0.487	0.125	**0.247**
	**Peak day**	0.054	0.005	0.010	0.134	0.012	0.018	0.021	0.042	0.088	0.016	**0.040**
	**Date peak**	29-May	28-May	11-May	14-May	23-Jun	6-Jun	12-May	5-Jun	21-May	24-Jun	**29-May**
**Pollen Allergen Potency × 10^−4^**		9.318	4.168	2.362	8.539	8.714	9.708	3.159	34.151	3.507	5.371	**8.705**
**Pollen risk**	**Moderate**	1	1	3	4	0	0	0	0	7	0	**1.6**
	**High**	0	0	0	0	0	0	0	0	0	0	**0**
**Ole e 1 risk**	**Moderate**	2	0	0	1	0	1	1	19	6	1	**3.1**
	**High**	1	0	0	3	0	0	0	0	2	0	**0.6**
**Pollen + Allergen**	**Moderate**	3	1	3	5	0	1	1	19	9	1	**4.3**
	**High**	1	0	0	3	0	0	0	0	2	0	**0.6**

Total Olea pollen (pollen/m^3^), pollen peak (pollen/m^3^), pollen peak date (days). Total allergen (ng/m^3^), allergen peak (ng/m^3^), allergen peak date (days) and Pollen Allergen Potency (ng/pollen). Number of days with allergy risk periods for pollen and allergen considering the thresholds of allergic risk for the pollen (Moderate 50–200 pollen/m^3^, High >200 pollen/m^3^; Galán et al., 2007), the allergens (Moderate 0.015–0.041 ng/m^3^, High >0.042 ng/m^3^) and both together.

Daily olive pollen concentrations and allergen values registered a great positive degree of association. A regression equation was developed to calculate aeroallergen thresholds for low, moderate and high symptomatology on hypersensitive patients (Ole e 1 = 0.00574 + 0.00018 × Pollen; R = 0.431; p < 0.000) following the threshold pollen concentrations recommended by the REA. Low allergen concentrations were considered between 0 and 0.014 ng/m^3^, Moderate between 0.015 and 0.041 ng/m^3^ and High when the Ole e 1 values exceed the 0.042 ng/m^3^ (**Table 1**). The obtained thresholds were used to determine the number of days with potential allergy hazard for sensitive patients. Considering pollen data, *Olea* only showed 5 years with days under moderate hazard of allergy (ranging from 1 to 7) and no days with high risk of allergy were registered during the period of study. When the amount of days under possible hazard of allergic symptoms due to airborne Ole e 1 concentrations was assessed, an increase of the risk days was detected. Moderate sensitization potential risk was registered during seven years (ranging from 1 to 19 days in 2016), and during three years some days with high hazard were observed (**Table 1**). When pollen and allergen concentrations were considered altogether to ascertain the real risk episodes, moderate hazard of allergy was detected during all study years, with the exception of 2013.

## Discussion

Pollen-soluble proteins have enzymatic activity essential for a wide range of functions of the male gametophyte physiology during germination processes as lipid transport, plant resistance, carbohydrate metabolism, expansins or proﬁlins (Radauer and Breiteneder, 2006). Ole e 1 allergens belong to the extensin family protein (Hu et al., 2014) and their biological function is related with pollen tube emergence, signalling and guidance, and pollen-stigma and pollen tube-style cells recognition processes (Rodríguez-García et al., 2003; Alché et al., 2004). Moreover, reactive oxygen species (ROS) produced by pollen-intrinsic NADPH oxidase activity during *Olea* pollen tube elongation is associated with the increases of allergic inflammatory response in sensitive people (Jimenez-Quesada et al., 2019; Mandrone et al., 2019).

Ole e 1 is recognized as one of the most important causes of Type-I respiratory allergy in the Mediterranean basin, after Poaceae pollen incidence (Davies et al., 2015). Their presence in the atmosphere is widely reported along the Mediterreanean basin and the boundary areas (Galán et al., 2013; Moreno-Grau et al., 2016; Plaza et al., 2016), intruding into the body through the upper airways to reach the mucosa (López-Rodríguez et al., 2016). Moisture and temperature conditions in the nasal mucosa of a sensitive patient are similar to the stigma conditions of a compatible female flower, which induces a great expression of Ole e 1 allergen prompting inﬂammatory disorders as an exacerbated T helper 2 (Th2)-type immune response against aeroallergens, usually inoffensive to most individuals (Akdis and Akdis, 2007). Studies conducted by Cariñanos et al. (2002) noted that Ole e 1 is quickly released from the pollen grain, feature that together with a high solubility, is considered as an important feature for a protein to be considered a major allergen (Cariñanos et al., 2002).

Analogous MPS duration is observed in our study compared with the reported in south Spain (Moreno-Grau et al., 2016) or in northern Portugal (Ribeiro and Abreu, 2014; Fernández-González et al., 2019). However, longer MPS with higher pollen annual amount is observed in Mediterranean regions as a consequence of the extensive olive crops (Aguilera et al., 2015). Previous studies demonstrated cross-reaction processes between the members of the Oleaceae family (Rodríguez-Rajo et al., 2010; Vara et al., 2016) which could extend the periods of Ole e 1-like proteins allergy. In north-western Spain sensitized people to olive pollen can also present allergic reactions during the winter, caused by *Fraxinus* pollen allergens, and during the early summer due to the *Ligustrum* flowering (Vara et al., 2016). Therefore, the Ole e 1 threshold values for pollinosis symptomatology could be reduced as consequence of the so-called “priming effect” (Connell, 1969). Increasing extension in hectares planted with olive trees throughout the south Galicia zones at the limit of the Mediterranean bioclimatical area prompted intensification in the atmospheric pollen concentrations during recent years.

Moreover, discrepancies between pollen appearance in the atmosphere and the period of symptomatology are detected (D´Amato et al., 2007; Galán et al., 2013) as a consequence of mismatches between the aeroallergen and pollen presence in the air (Rodríguez-Rajo et al., 2011; Plaza et al., 2016). Some research papers pointed out that olive pollen counts are not representative of exposure to its main allergen Ole e 1 (Galán et al., 2013; Vara et al., 2016). Although pollen allergens are firstly carried by pollen grains (D’Amato, 2001), they may also be transported in the microaerosol suspension smaller than pollen grains, which could remain longer periods in the atmosphere. Our study detected some previous allergen peaks and other Ole e 1 peaks under low pollen concentrations in the bioaerosol. Some authors proposed different pathways for this Ole e 1 release, such as from pollen wall, through the aperture regions under pollen hydration, or from rests of tapetal cells during anthesis (Alché et al., 2004; Rodríguez-Rajo et al., 2010). In addition, rapid metabolic activation for germination and pollen tube development occurred because olive pollen hydrates quickly under certain degrees of humidity, could be the source of increases in aeroallergen concentration during previous periods of pollen grains presence. Our study showed Ole e 1 increases in the atmosphere after rainfall episodes. The classically information for hyper sensitized patients is the concentration of pollen grains in the atmosphere and their temporal distribution. The number of episodes with moderate and high allergen risk for allergic symptomatology increased when the Ole e 1 data was considered. The development of new advanced methods for the determination of the real allergenic load in the air is required to complement the classic pollen counts to improve and optimize the prescription of medical treatments (Rodríguez-Rajo et al., 2011; Fernández-González et al., 2019).

Furthermore, our results showed that pollen concentrations and allergen potency of olive pollen was lower in the Northern Spain area than in the Mediterranean basin, also due to the expansion of the olive tree cultivation (Galán et al., 2013; Moreno-Grau et al., 2016; Plaza et al., 2016; Vara et al., 2016). The study of pollen potency is very important to know the real allergenic load in the atmosphere, as previous researches noted that pollen counts did not reproduce real allergen exposure (Galán et al., 2013). Yearly *Olea* pollen values in southern Spain are 2 and 8 times higher than in the central and northern areas, respectively, while rates increase considerably to seven and 40 times when allergen exposure was assessed (Galán et al., 2013; Moreno-Grau et al., 2016). Differences in pollen allergen potency reported across Europe may be related with specific local conditions, long-range transport and other factors. High-potency *Olea* pollen can be transported over 400 km resulting in increases of 40% of the olive exposure (Galán et al., 2013).

Finally, inverse relationships between *Olea* pollen counts and Ole e 1 concentrations can reflect an alternate bearing behaviour (Moreno-Grau et al., 2016), modulated by year-to-year meteorological oscillations (Fernández-Caldas et al., 2007). Our research detected some years of low annual pollen concentrations and high total Ole e 1 amounts (as ex. during the years 2009, 2012 and 2017). The tree seems to reflect a higher ability for the allergen Ole e 1 expression in years with lower pollen production to ensure an appropriate pollen germination and tube elongation to a successful fertilization process. This fact could justify higher sensitization rates in years with lower expected pollen concentrations. *Olea* tree is well recognized for its tendency towards an alternative bearing behaviour pattern for several biological features (Erel et al., 2013; Moreno-Grau et al., 2016).

The present study contributes to the improvement of the detection of the risk of allergy to *Olea*, one of the most important allergenic pollen types in the Mediterranean basin region, analysing a secondary impact of the Ole e 1 protein whose main biological function was related to the development of the pollen tube. The determination of the real allergenic load in the atmosphere complements traditionally available information of airborne pollen concentrations, offering a new more accurate perspective of pollen allergy phenomenon and improving the health system capacity to protect sensitized population. The information generated supposes a valuable tool for reducing the consumption of medications, since the worst clinical symptoms of pollinosis can be prevented before its appearance by alerting people to the different risk levels detected in the atmosphere.

## Data Availability Statement

The raw data supporting the conclusions of this article will be made available by the authors, without undue reservation.

## Author Contributions

MF-G, EG-F, and FR-R conceptualized and designed the experiments. MF-G, DF-G, and FR-R acquired, analyzed, and interpreted the data. FR-R and MF-G drafted the manuscript. EG-F and DF-G contributed to critical revision of the manuscript.

## Funding

This research was funded by the BV1 Reference Competitive Research Groups ED431C 2017/62 (Xunta de Galicia, Spain), and the CITACA Strategic Partnership ED431E 2018/07 (Xunta de Galicia, Spain).

## Conflict of Interest

The authors declare that the research was conducted in the absence of any commercial or financial relationships that could be construed as a potential conflict of interest.
